# Last Steps as a Trainee, First Steps as a Consultant Specialist

**DOI:** 10.1192/j.eurpsy.2022.193

**Published:** 2022-09-01

**Authors:** C. O’Loughlin

**Affiliations:** Health Education England, East Of England, Cambridge, United Kingdom

## Abstract

The transition from being a senior trainee to a qualified specialist presents a variety of challenges under normal circumstances, with the pandemic further disrupting the experience of trainees. This presents an environment where the acquisition of key competencies, both clinical skills and wider capabilities, becomes more demanding for trainees and where training organisers need to re-evaluate their programmes to consider the different opportunities available. This introduction to the interactive session will consider how the experience in the United Kingdom led to new ways of delivering training, the challenges faced and consider the key skills new specialists need to be effective clinical leaders as they move into a senior role.

**Disclosure:**

No significant relationships.

